# Regulatory T Cells in GVHD Therapy

**DOI:** 10.3389/fimmu.2021.697854

**Published:** 2021-06-18

**Authors:** Wen-wen Guo, Xiu-hua Su, Ming-yang Wang, Ming-zhe Han, Xiao-ming Feng, Er-lie Jiang

**Affiliations:** ^1^ State Key Laboratory of Experimental Hematology, Institute of Hematology and Hospital of Blood Disease, Chinese Academy of Medical Sciences and Peking Union Medical College, Tianjin, China; ^2^ School of Medicine, Cheeloo College of Medicine, Shandong University, Jinan, China

**Keywords:** regulatory T cells, acute graft *versus* host disease, chronic graft *versus* host disease, hematopoietic stem cell transplantation, adoptive cellular therapy

## Abstract

Graft *versus* host disease (GVHD) is a common complication and the leading cause of morbidity and mortality after allogeneic hematopoietic stem cell transplantation (allo-HSCT). Pharmacological immunosuppression used in GVHD prophylaxis and treatment lacks specificity and can increase the likelihood of infection and relapse. Regulatory T lymphocytes (Tregs) play a vital role in restraining excessive immune responses and inducing peripheral immune tolerance. In particular, clinical trials have demonstrated that Tregs can prevent and treat GVHD, without increasing the risk of relapse and infection. Hence, adoptive transfer of Tregs to control GVHD using their immunosuppressive properties represents a promising therapeutic approach. To optimally apply Tregs for control of GVHD, a thorough understanding of their biology is necessary. In this review, we describe the biological characteristics of Tregs, including how the stability of FOXP3 expression can be maintained. We will also discuss the mechanisms underlying Tregs-mediated modulation of GVHD and approaches to effectively increase Tregs’ numbers. Finally, we will examine the developing trends in the use of Tregs for clinical therapy.

## Introduction

Allogeneic hematopoietic stem-cell transplantation (allo-HSCT) is a curative therapy for patients with many hematological malignancies; however, graft *versus* host disease (GVHD) is a major obstacle to the utility of allo-HSCT as it contributes to subsequent mortality and morbidity. GVHD can be classified into acute (aGVHD) and chronic (cGVHD) forms and is characterized by attack of host tissues by donor lymphocytes, owing to disparities in major histocompatibility complex (MHC) molecules or minor histocompatibility antigens (mHAs), which elicit an immune response. Although the immunosuppressive agents, cyclosporine and methotrexate, are administered after allo-HSCT as prophylaxis, aGVHD incidence rates range from 20 to 80% (1), while 6 to 80% of patients develop cGVHD. Glucocorticoids are the first-line treatment for GVHD (1–3); however, only 50–80% and 40–50% of patients with aGVHD and cGVHD, respectively, respond to steroid therapy (1, 2). If patients are resistant to glucocorticoids, overall survival rates are dismal at only 5 to 30% (4). At present, there is no consensus on standard second-line treatment. This situation emphasizes the need for development of innovative therapeutic strategies to control pathological immune responses following allo-HSCT.

In recent years, with increased understanding of regulatory cell populations, cellular therapy, particularly adoptive transfer of CD4^+^CD25^+^ regulatory T cells (Tregs), has attracted more attention (5). In 1995, Sakaguchi (6) first identified a population of CD4^+^ cells expressing high levels of IL-2 *α*-chain receptor (CD25) in lymph nodes and spleens of BALB/c nu/+ mice, which could protect thymectomized mice from autoimmune disease and contribute to maintaining peripheral immune tolerance by suppressing immune responses against self- or non-self-antigens. Later, transcription factor forkhead box P3 (FOXP3) was identified as specifically expressed in Tregs and as making an indispensable contribution to controlling Treg development and suppressive function (7), deepening the understanding of this cell subset. The IL-7 receptor, CD127, is a biomarker inversely correlated with FOXP3 expression and Tregs’ inhibitory activity (8). Since FOXP3 is expressed intracellularly, the combination of CD4, CD25, and CD127 has been widely used to isolate Tregs and CD4^+^CD25^+^CD127^–^ T cells are now referred to as Tregs. In addition to self-tolerance, Tregs also have a vital role in inducing tolerance of alloantigens (9–11). at url breaking operator Taylor et al. (9) found that CD25-depleted CD4^+^ cells showed increased responsiveness to allogeneic antigens and that addition of CD4^+^CD25^+^ cells had a potent capacity to regulate CD4^+^ T cell alloresponses. Meanwhile, CD4^+^CD25^+^ cells were found to induce transplantation tolerance in experiments involving transfer of CD4^+^CD25^+^ Tregs from primarily tolerant mice to syngeneic recipients, which protected recipients from graft at url breaking operator rejection (10, 11). Based on the suppressive capacity of CD4^+^CD25^+^ Tregs toward alloreactive T cells, Cohen et al. (12) demonstrated that removal of CD4^+^CD25^+^ Tregs from the graft during transplantation accelerated the occurrence of GVHD, while addition of freshly isolated or *ex vivo*-expanded CD4^+^CD25^+^ Tregs could delay or even prevent GVHD after allo-HSCT. Tregs were found to prevent GVHD for a long time due to their survival and expansion *in vivo* after transplantation in bone marrow transplantation models (13). Besides, adoptive transfer of Tregs could accelerate the immune reconstitution after transplantation, owing to the prevention of damage of the thymic and secondary lymphoid microenvironment caused by GVHD, which was important for T cell immunity (14). Given these properties of suppressing excessive allogeneic responses, cellular therapy, based on adoptive transfer of Tregs to control GVHD has been the focus of study (12, 15–18).

T cells with immunosuppressive function are a diverse group of cells. Except for CD4^+^ Tregs, it has been confirmed that CD8^+^ Tregs can regulate excessive immune responses to control GVHD in animal models as well (19). Being different from CD4^+^ Tregs, the characteristics of CD8^+^ Tregs are controversial, and specific surface markers to isolate them have not reached agreement (20). In addition to *αβ* T cells, *γδ* T cells are also capable of immunoregulation (21). Regulatory *γδ* T cells (*γδ* Tregs) function to regulate GVHD, which can be induced by granulocyte colony-stimulating factor (G-CSF) (22). In this review, we will focus on CD4^+^CD25^+^FOXP3^+^ Tregs.

Tregs have the potential to attenuate GVHD without impairing the graft *versus* leukemia (GVL) effect significantly, making the adoptive transfer of Tregs a promising strategy for treatment of GVHD (23, 24); however, translation of this phenomenon into clinical application continues to face numerous challenges, particularly the instability of Tregs and the difficulties in obtaining sufficient quantities of cells to transfer. In this review, we discuss the biological characteristics of Tregs administered in the context of GVHD. Furthermore, we focus on how to solve the problem of insufficient Treg numbers for adoptive transfer and discuss the clinical prospects for application of Tregs.

## The Theoretical Basis of Tregs’ Administration to Treat GVHD

### Tregs’ Definition and Function

Tregs account for only 5–10% of CD4^+^ T cells in peripheral blood, but they are essential for maintenance of immunological tolerance (6, 9–12). FOXP3 is specifically expressed in Tregs and acts as a major regulator that controls their development and stability (7, 25). Inactivating mutations or specific deletion of FOXP3 causes a lethal autoimmune syndrome due to a deficiency of Tregs (7). Tregs can be roughly divided into two groups according to their developmental origin. First, thymic Tregs (tTregs), also known as nTregs, are generated when CD4^+^ single-positive thymocytes encounter self-antigen stimuli in the thymus during development; T cell receptors (TCRs) expressed on tTregs mainly recognize self-antigens, which means tTregs have advantages in preventing autoimmune disease (26, 27). The other type of Tregs develops from naïve CD4^+^ T cells in the periphery following antigen encounter, through exposure to appropriate cytokines, such as transforming growth factor-*β* (TGF-*β*) and IL-2 (28–31). When this pathway occurs *in vivo*, the resulting FOXP3^+^ Treg cells are referred to as peripherally induced Tregs (pTregs), whereas, when it takes place *in vitro*, they are termed induced Tregs (iTregs) (28–31). Relative to tTregs, pTregs are considered to play an important role in maintaining mucosal tolerance, as the TCRs expressed on pTregs can also be specific for foreign antigens from commensal bacteria (32). In mice, neuropilin-1 (NRP1) is selectively expressed on nTregs rather than pTregs, whether they are generated *in vivo* or *in vitro*, and can be used to distinguish nTregs from pTregs (33). Based on analysis of NRP1, Yadav et al. (33) found that NRP-1^lo^ Tregs have similar ability to suppress autoimmune responses as NRP-1^hi^ Tregs, but that the function of NRP-1^lo^ Tregs was compromised in inflammatory and lymphopenic environments, relative to that of NRP-1^hi^ Tregs. Unfortunately, no cellular markers have been found to distinguish nTregs and pTregs in humans. Currently, the evaluation of Treg-specific demethylated region (TSDR), a conserved CpG-rich region within the FOXP3 locus, is the only way to distinguish nTregs, and the stability of FOXP3 expression is positively correlated with DNA demethylation at the TSDR (34). The TSDR is completely demethylated in nTregs, but iTregs exhibit incomplete demethylation of TSDR and are prone to losing FOXP3 expression and suppression ability (34), which may explain their instability in inflammatory environments.

### FOXP3 Stability in Tregs

FOXP3, which is regarded as a Treg lineage-specific factor, acts as a master regulator of gene expression in Tregs and exerts regulatory functions at the transcriptional, epigenetic, and post-transcriptional levels (35). Continuous FOXP3 expression is indispensable for maintenance of the Tregs’ immunosuppressive phenotype; however, under certain proinflammatory conditions, Tregs are prone to losing FOXP3 expression and transdifferentiating into pathogenic T cells, also referred to as ex-Tregs (36). Therefore, it is particularly important to enhance FOXP3 expression in Tregs to ensure that they continue to exert immunosuppressive activity under post-transplantation conditions. Our laboratory and others found that liver kinase b1 (LKB1) is essential for maintaining the stability of FOXP3 expression and the suppression capacity of Tregs in murine models by deletion of the gene encoding LKB1 specifically in Tregs (37–40). We also found that LKB1 may prevent STAT4-mediated methylation of the TSDR, ensuring stable FOXP3 expression (37). Furthermore, we observed dramatically decreased expression of FOXP3 in human Tregs owing to knockdown of the *LKB1* gene (40). MicroRNAs (miRNAs) can govern the expression of protein-coding genes at the post-transcriptional level (41). Cobb et al. found that miRNA profiles expressed in Tregs were distinct from those in conventional CD4^+^ T cells and confirmed that eliminating miRNAs by conditional deletion of Dicer (an RNAse III enzyme needed to generate functional miRNA) could influence the development of Tregs in the thymus, reduce the number of Tregs in the periphery, and down-regulate FOXP3 expression (42). Moreover, mice lacking Dicer were prone to developing immune pathology (42). Nevertheless, the detailed mechanisms underlying interactions between miRNAs and FOXP3 are not fully understood. MiR-146b can restrain FOXP3 protein levels by targeting TNF receptor-associated factor 6 (TRAF6) and suppressing the NF-*κ*B pathway (43). Conversely, MiR-146b antagomirs function to promote the proliferation and suppressive ability of Tregs (43). Further, miR-4281 interacts directly with the TATA-box motif in the FOXP3 promoter, thereby strongly increasing FOXP3 expression (44). Similarly, miR-142-3p knockdown upregulates FOXP3 expression and enhances the anti-apoptotic and suppressive function of Tregs (45). Ectopic expression of FOXP3 can confer partial Tregs miRNA profiles, demonstrating that FOXP3 may control Treg-specific miRNA expression (42). Hence, miRNAs may have important roles in regulating Tregs’ biology and potentially represent strong targets for intervention against GVHD.

### Characteristics of GVHD

The clinical manifestations of aGVHD include an exaggerated inflammatory response, usually involving the skin, intestine, and liver, which occurs within the first 100 days after allo-HSCT (46). Ferrara categorized the occurrence of aGVHD into three continuous phases (47): First, the activation of antigen presenting cells (APCs) by proinflammatory cytokines and danger-associated molecular pattern molecules (DAMPs), which originate from damaged host tissues in response to primary disease and conditioning regimens (47, 48); Second, after encountering activated APCs, donor T cells are rapidly expanded and differentiated into effector T cells (Teffs); Finally, Teffs migrate to target organs with the help of chemokines, causing further damage to host tissues (47, 48). Compared with aGVHD, the mechanisms underlying cGVHD are not clearly understood. The manifestations of cGVHD are similar to those of autoimmune diseases, involve more organs than aGVHD, and usually develop more than 100 days after allo-HSCT (47). Overall, GVHD can be considered as an imbalance between the effector and regulatory arms of the immune system and is characterized by an overproduction of inflammatory cytokines (49, 50).

Numbers of Tregs in the peripheral blood and target organs decline in the inflammatory environment of GVHD, which, in turn, increases GVHD severity (51, 52). Conversely, when Tregs are co-transferred in equal numbers with CD4^+^ T cells, GVHD can be modestly inhibited in animal models (53). Furthermore, improving early reconstitution of Tregs can prevent GVHD by inhibiting the rapid oligoclonal proliferation of pathogenic CD4^+^ Teffs (54). Understanding the mechanisms underlying immune tolerance induction by Tregs can provide information about potential therapeutic targets of GVHD.

### Role of Tregs in GVHD

**Figure 1:**
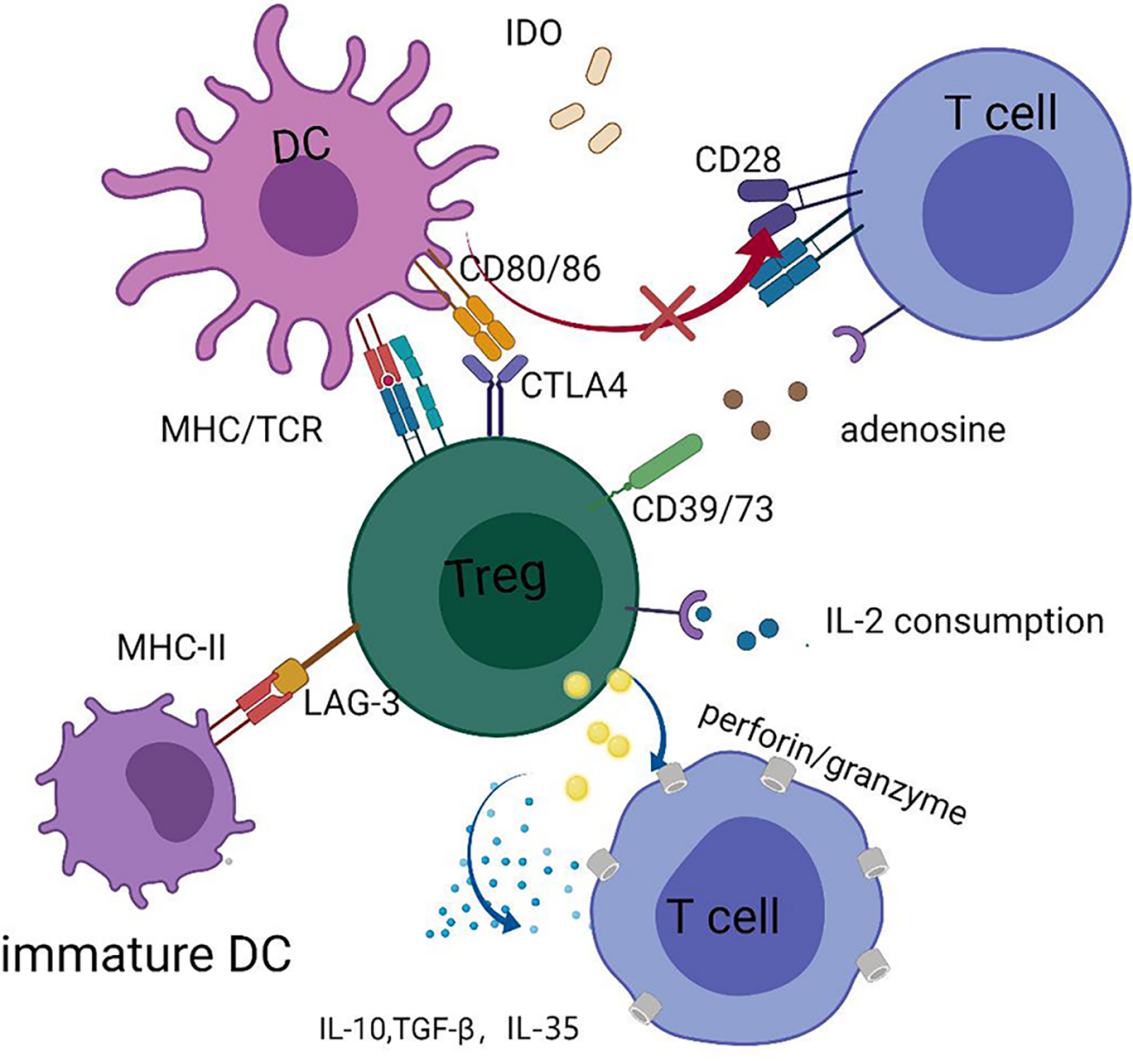
Tregs can secrete perforin and granzyme B to kill Teffs directly and secret IL-10, IL-35, and TGF-*β*, inhibitory cytokines to suppress functions of Teffs; Tregs can cause IL-2 starvation of Teffs *via* highly expressing CD25. Tregs exert the function of suppression by interaction with DCs, such as downregulating the expression of CD80/CD86 in DCs and interfering with the maturation of DCs. Tryptophan is vital for the survival of Teffs and Tregs can enhance the expression of indoleamine 2, 3-dioxygenase (IDO) in DCs, which accelerates the decomposition of tryptophan. Adenosine triphosphate (ATP) is a pro-inflammatory factor, and CD39/73 expressed on Tregs can transform ATP to adenosine, an anti-inflammatory factor.

In general, Tregs can regulate and suppress excessive responses to alleviate GVHD using both contact and non-contact-dependent mechanisms, which can be classified into four categories: cytolysis, secretion of inhibitory cytokines, metabolic disruption, and targeting of dendritic cells (DCs) (55) (**Figure 1**). Perforin and granzyme B secreted by Tregs can kill Teffs directly (55). Furthermore, inhibitory cytokines, such as interleukin-10 (IL-10), IL-35, and TGF-*β*, which are expressed by Tregs, are required for Tregs’ function (48, 55–57). IL-2 is indispensable for the homeostasis of both Tregs and Teffs. Tregs consume high amounts of IL-2 in local sites, since CD25 (the IL-2 receptor alpha chain) is highly expressed by Tregs, and this may lead to IL-2 starvation of Teffs (48, 55). The reciprocal relationship between Tregs and DCs performs a vital and complex function in controlling GVHD. In addition to killing reactive T cells *via* cell–cell contact, Tregs can act on multiple target cells, particularly DCs. Moreover, Tregs appear to have a more stable association with DCs than that with CD4^+^ T helper (T_H_) cells in NOD mouse models, which prevents subsequent interaction between DCs and T_H_ cells (58). Thus, the interaction between Tregs and DCs may have a core role in the mechanisms underlying Tregs-mediated immune suppression (58, 59). DCs are considered the most powerful APCs and have a dual role in GVHD development (60–65); host DCs or *de novo* generated donor DCs both contribute to T-cell priming by presenting alloantigens in the context of HLA molecules, as well as providing secondary signals to promote full T cell activation (60–63). Tregs constitutively express cytotoxic T-lymphocyte antigen 4 (CTLA-4), the affinity of which for CD80/86 expressed on DCs is superior to that for CD28, hindering complete T-cell activation *via* blocking the binding of CD28 and CD80/86 (66). Further, Tregs can facilitate the removal and degradation of CD80/86 from DCs *via* CTLA-4 through the process of trans-endocytosis (67). In addition, immunosuppressive cytokines, such as IL-10, released by Tregs can interfere with DC activation and antigen presentation (68). Tregs can hamper DC maturation, rendering them deficient in priming T cell activation, and lymphocyte activation gene 3 (LAG-3), expressed by Tregs, may play a dominant role in the process of DC maturation (69–72). Recently, Mavin et al. (70) discovered that human Tregs can modulate DC function through disturbing their reprogramming during maturation, which may involve the NF-*κ*B signaling pathway and WNT5A expression. In addition to influencing the activity of DCs themselves, Tregs may alter DC function; for example, Treg-treated DCs tend to skew CD4^+^ naïve T cell polarization and impair CD8^+^ Teffs function in inducing GVHD (70). In contrast, DCs can induce donor T cell tolerance by promoting the expansion and function of Tregs, thus protecting them from GVHD (60, 73). Our laboratory found that LKB1 has an important role in DCs to increase the number of Tregs, which has also been confirmed in other studies (74–76). Granulocyte-macrophage colony-stimulating factor (GM-CSF) functions to increase CD4^+^CD8^–^ DC numbers, thus preferentially inducing Tregs expansion, and alleviating tissue damage in a cGVHD mouse model (77). The relationship between DCs and Tregs intricately controls GVHD, and further research is warranted to inform the development of new strategies to prevent GVHD.

### Tregs in GVL

T cells originating from donors can eliminate remaining tumor cells, hence allo-HSCT is considered the only curative therapy for numerous malignant hematological diseases. This also means that immunosuppressive Tregs controlling GVHD by inhibiting the initial activation of alloreactive T cells may compromise the GVL effect, thereby increasing the risk of relapse and infection. Experiments in several animal models have revealed that Treg therapy can suppress GVHD while maintaining GVL, thus separating GVHD from GVL (78, 79). This may involve Treg-mediated inhibition of excessive donor T cell proliferation and downregulation of serum proinflammatory cytokine levels, while not interfering with the activation of conventional T cells (Tcons), particularly the ability of CD8^+^ T cells to kill tumors (78); however, some preclinical experiments have also demonstrated that CD4^+^ iTregs can partially impair GVL in a mouse model, with animals suffering short-term leukemia relapse (80, 81). The combination therapy of CD4^+^ iTregs and CD8^+^ iTregs may provide a new way to solve the problem because the GVL effect can be preserved by CD8^+^ iTregs, and CD4^+^ iTregs function to attenuate GVHD in the meanwhile, which achieves the effect of one plus one being greater than two (81). Excitingly, Treg-based therapy has seldom been found to have a detrimental influence on the risk of relapse and infection in clinical trials (18, 82–84).

## Tregs’ Expansion for GVHD Therapy

### Expansion *Ex Vivo*

**Figure 2:**
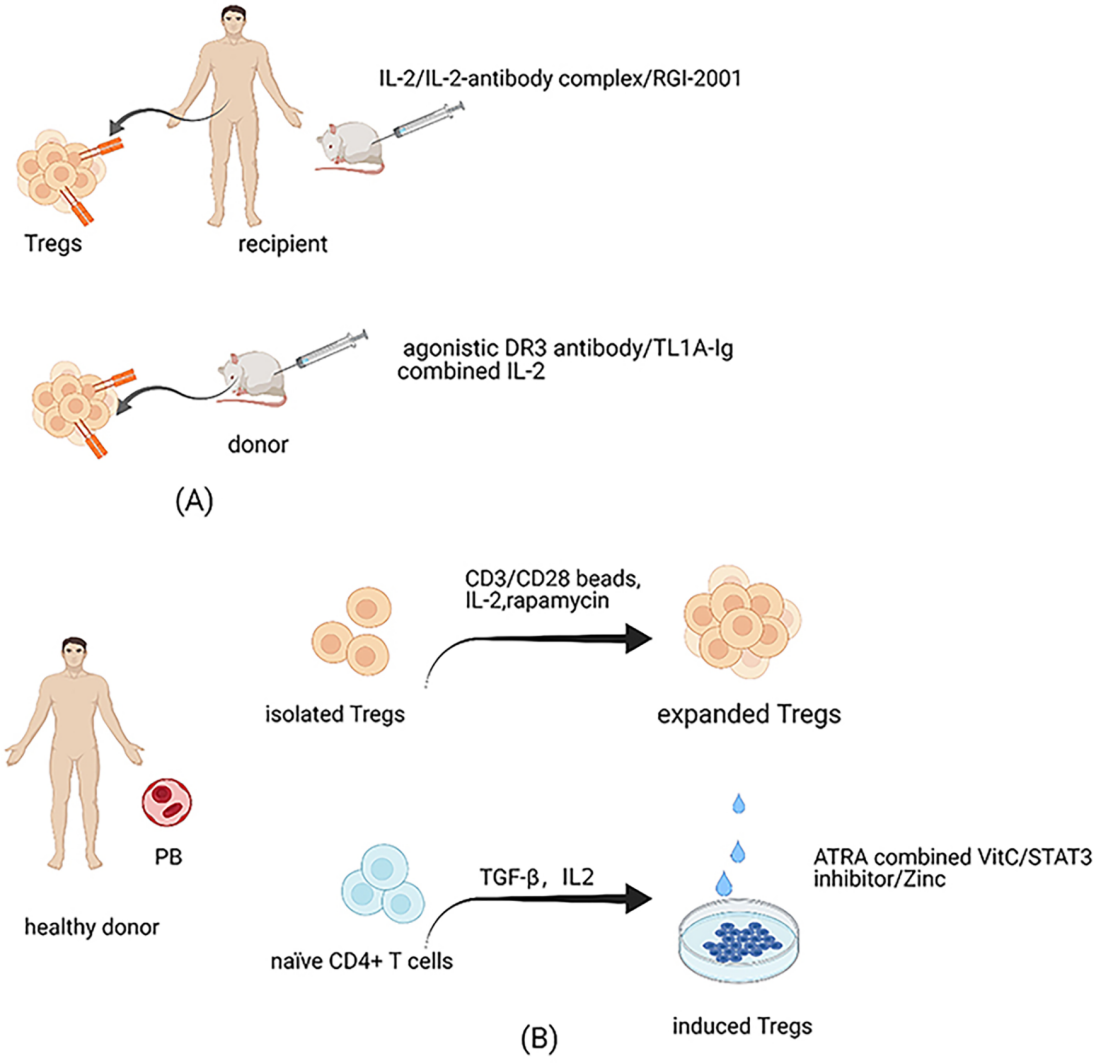
Treg expansion. **(A)** Expansion of Tregs in recipients and donors. Administration of low-dose IL-2 can expand Tregs of hosts preferentially *in vivo* since a high-affinity IL-2 receptor is expressed highly on Tregs. Considering IL-2 can also enhance the activation of Teffs, antibody-IL-2 complex is created, which can electively expand Tregs *in vivo* due to altering the structure of IL-2. Besides, RGI-2001 also has the function to accelerate the expansion of Tregs *in vivo*. The effectiveness of these cells has been tested in human and animal models. Reagents to expand Tregs of donors have been administrated in animal models, such as agonistic antibody against *α*DR3 and TL1A-Ig combined with IL-2. **(B)** Expansion of Tregs isolated from donors *in vitro* and methods to improve iTregs’ stability. Tregs isolated from healthy donors and expanded *in vitro* are the most common source at the expense of complex technologies and costly prices. Because of the unstable properties of iTregs, methods have been tried to improve its stability, such as the combination of all-trans retinoic acid and vitamin C, administration of STAT3 inhibitor, and the supplementation of Zinc.

As mentioned above, Tregs account for a small proportion of CD4^+^ T cells in the peripheral blood. Therefore, the numbers of Tregs freshly isolated from donors are far from sufficient to cater for clinical infusion requirements. Furthermore, the purity of Tregs isolated from donors is sub-optimal, as there is a lack of specific markers to distinguish Tregs from Tcons. Currently, Tregs isolated by leukapheresis from healthy donors and expanded by exposure to *α*CD3/*α*CD28 beads and IL-2 stimulation are a common source for cellular therapy in the clinic (85) (**Figure 2**). Tregs expanded by using good manufacturing practices–compatible protocol were confirmed to prevent GVHD and retain GVL effectively in animal models (86). The approach of automated clinical-grade expansion of Tregs *ex vivo* has been developed to improve the purity and quantity of the infused Tregs (85, 87). Nevertheless, the technology to isolate and expand Tregs is complex and costly; hence, new strategies to produce sufficient functional Tregs are required.

Although iTregs are intrinsically unstable, adoptive transfer of iTregs may also function to control GVHD (53, 88, 89). Notably, iTregs can overcome the problem of lack of sufficient cell quantity, as they can be manufactured abundantly *ex vivo*. Further, alloantigen-specific iTregs are considered more effective than polyclonal Tregs as they directly target specific antigens (29, 88). Nevertheless, iTregs are prone to lose FOXP3 expression, particularly under inflammatory conditions, which limits their therapeutic activity in GVHD (29, 90). There have been attempts to optimize iTregs’ stability (**Figure 2**). Kasahara et al. (88) found that stable iTregs could be generated using a combination of all-trans retinoic acid and vitamin C. Vitamin C-treated iTregs significantly alleviated GVHD symptoms in murine models compared with untreated iTregs. Further, iTregs treated with STAT3 inhibitor exhibited dramatically enhanced suppression ability and achieved stability *via* high levels of FOXP3 demethylation (91). Furthermore, STAT3 phosphorylation-inhibited iTregs have potent function in preventing xenogeneic GVHD by reducing excess immune responses caused by alloreactive T cells (92). Zinc supplementation may also increase iTreg numbers while maintaining their stability by prolonging FOXP3 expression (93). Enhancing iTregs’ stability will solve the problem of insufficient Treg quantity for the requirements of clinical application.

### Expansion *In Vivo*

Fujioka et al. (94) found that a low ratio of Tregs to CD4^+^ T cells in the early stage after allo-HSCT can predict impending aGVHD. Our laboratory results also suggest that Tregs from patients with aGVHD become defective in terms of their stability, survival, and suppressive function (40). Expansion of Tregs in recipients after allo-HSCT may be a promising strategy to alleviate GVHD (**Figure 2**). It is established that IL-2 is vital for the development, proliferation, and activity of Tregs (95), which can react to low concentrations of IL-2 by expressing high levels of a high-affinity IL-2 receptor (CD25). Administration of low-dose IL-2 in cGVHD patients was associated with preferential expansion of Tregs *in vivo* and attenuated the symptoms in certain patients (96, 97). Notably, IL-2 can also target many other cells, including promoting conventional T cell activation, and the appropriate effective dose for controlling GVHD is difficult to manage (95). Trotta et al. (98) generated a human antibody against IL-2 and combination with the antibody altered IL-2 conformation. The antibody–IL-2 complex could considerably and selectively expand Tregs *in vivo* and was effective in protecting mice from GVHD. Recently, Hirai et al. (99) innovatively engineered an orthogonal IL-2/IL-2 receptor (IL-2R) pair. The results demonstrated that transduced Tregs with orthogonal IL-2R were selectively expanded by orthogonal IL-2 stimulation without increasing other T cell subsets in murine models. Besides, this method did not reduce the function of Tregs and improved graft tolerance. Infusion of RGI-2001, a liposomal formulation carrying *α*-galactosylceramide dramatically improved the survival of mice with aGVHD by enhancing the expansion of alloantigen specific CD4^+^CD25^+^ Tregs *in vivo*; notably, administration of RGI-2001 did not abrogate GVL (100). Further, a clinical trial of RGI-2001 showed that the incidence of severe GVHD was lower in responders than in non-responders (101).

In addition to expanding Tregs in hosts, methods to increase the number of donor Tregs for transfer *in vivo* have been attempted (**Figure 2**). An agonistic antibody against death receptor 3 (*α*DR3) has been found to enhance CD4^+^FOXP3^+^ Treg proliferation *in vivo* when administered to the donor, thus increasing the percentage of Tregs in the graft (102–104). Further, *α*DR3-treated Tregs demonstrated potent proliferation and suppression abilities when transferred to host mice. The severity of GVHD in recipient mice receiving grafts from *α*DR3-treated donors was significantly less than that in isotype-treated donors (102–104). Similarly, TL1A-Ig (a soluble fusion protein from the natural ligand of tumor necrosis factor superfamily receptor 25 (TNFRSF25)) combined with IL-2 led to marked Treg expansion in donors *via* respective targeting of TNFRSF25 and CD25 *in vivo*. Adoptive transfer of Tregs from donors treated with TL1A-Ig/IL-2 effectively protected recipients from GVHD (16, 17). Furthermore, preclinical trial data suggest that GVHD after allo-HSCT can be controlled in patients with low numbers of Tregs stimulated by TL1A-Ig combined with IL-2, as this method of amplification can enhance Tregs’ suppressive activity (16). Reagents found to increase Tregs’ numbers and promote their inhibitory function in the donor may be used to develop new strategies for expanding Tregs *ex vivo* in the future.

## New Sources of Tregs for GVHD

### Third-Party Tregs

In animal models and clinical trials, donors have been the most common source of Tregs used to modulate GVHD. As CD4^+^CD25^+^FOXP3^+^ Tregs are scarce in the peripheral blood and HLA-matched donors are not always available, the efficacy and safety of Tregs from other sources have been evaluated for use in GVHD control (105). Tregs derived from a third-party [umbilical cord blood (UCB)] could confer protection from GVHD in a xenogeneic GVHD mouse models (106). Mice prophylactically injected with UCB-derived Tregs achieved better GVHD scores and overall survival rates than those receiving peripheral blood mononuclear cells (PBMCs) only (106). Another study found that Tregs derived from third-party mice could inhibit GVHD development compared with those originating from donors or hosts. Although the optimal therapeutic effect was observed in mice treated with donor Tregs, third-party Tregs could still be considered as a promising alternative source (105). Large numbers of Tregs can be isolated from pediatric thymuses, which are generally removed during cardiac surgery (107). Expanded thymic Tregs manifest stable FOXP3 expression and even maintain inhibitory ability under inflammation conditions. Furthermore, thymic Tregs more effectively protected mice from GVHD than Tregs derived from peripheral blood in xenogeneic GVHD mouse models. Therefore, pediatric thymuses may become an alternative source of functional Tregs (107).

### CAR Tregs

Compared with polyclonal Tregs, alloantigen-specific Tregs, particularly those with chimeric antigen receptors (CARs), have the advantage of being able to achieve specific immunosuppression using fewer cells (108). CAR technology confers Tregs with superior ability to identify whole proteins expressed in cells, dispensing with HLA molecule restriction. MacDonald et al. (108) created alloantigen-specific Tregs using a CAR targeting HLA-A2, which is commonly mismatched in transplantation, and the A2-CAR Tregs maintained phenotypic stability and suppressive function. Surprisingly, the A2-CAR Tregs prominently delayed GVHD development and improved mouse survival compared with polyclonal Tregs in xenogeneic GVHD models. Further, owing to their larger numbers in circulation and considerable proliferation rates, CD4^+^ T cells can be transduced to express both A2-CAR and FOXP3 (109). These genetically modified CD4^+^ T cells (A2-CAR/FOXP3 CD4^+^ Tregs) obtained a stable inhibitory phenotype and could suppress inflammation responses. Further, A2-CAR/FOXP3 CD4^+^ Tregs could significantly alleviate inflammatory pathology in a GVHD mouse model (109).

Third-party and engineered Tregs raise the prospect of universal Tregs, which could be provided as off-the-shelf products for use in patients (110). Universal donor Tregs can be manufactured to reduce immunogenicity by knocking out classical HLA molecules and knocking in non-canonical HLA molecules, such as HLA-E or HLA-G, thus escaping host immune system recognition (110). With advances in genome editing technology, induced pluripotent stem (iPS) cells may become a potential source of universal donor Tregs, with optimal immunosuppressive activity after engineering (110). Adoptive therapy of universal donor Tregs may represent a promising approach for patients with GVHD.

## Tregs Application Strategies in GVHD

As mentioned above, Tregs play an essential role in maintaining immunological tolerance. Therefore, adoptive transfer of Tregs to directly reduce the incidence or severity of GVHD after allo-HSCT has been the focus in the field of transplantation. Main clinical trials have been summarized in **Table 1**. What is more, increasing the frequency and stability of Tregs *in vivo* by reagents, such as IL-2, to control excessive immune responses has also aroused interest.

**Table 1:** Main clinical trials with Tregs in stem cell transplantation.

Number of patients	Phase	Type of transplantation	Sources of Tregs	Dosage of Tregs	Indication	Status	Trial ID
28	II	Haplo-HSCT	donor Tregs	2 × 10^6^/kg or 4 × 10^6^/kg	GVHD prevention	Recruiting	(82), Di Ianni et al. NCT03977103
23	I	UCBT	UCB Tregs	0.1–30 × 10^5^/kg	GVHD prevention	Completed	(83), Brunstein et al. NCT00602693
43	II	Haplo-HSCT	donor Tregs	Mean 2.5 × 10^6^/kg	GVHD prevention	Recruiting	(84), Martelli et al. NCT03977103
33	I	UCBT	UCB Tregs	3–100 × 10^6^/kg	GVHD prevention	Completed	(23), Brunstein et al. NCT00602693
12	I/II	MSD allo-HSCT	donor Tregs	1× 10^6^/kg–3× 10^6^/kg	GVHD prevention	Recruiting	(24), Meyer et al. NCT01660607
16	I	MSD allo-HSCT	iTregs	3× 10^6^/kg–10× 10^8^/kg	GVHD prevention	Completed	(89), MacMillan et al. NCT01634217
50	II	HLA-haploidentical HSCT	donor Tregs	2 × 10^6^/kg	GVHD prevention	Recruiting	(18), Pierini et al. NCT03977103
35	II	allo-HSCT	donor Tregs	≥0.5× 10^6^/kg	Steroid refractory cGVHD	Recruiting	NCT01903473
20	I/II	allo-HSCT	donor Tregs	5× 10^5^/kg, 1× 10^6^/kg, 2 × 10^6^/kg	Severe refractory cGVHD	Recruiting	NCT02749084

Allo-HSCT, allogeneic hematopoietic stem cell transplantation; Haplo-HSCT, haploidentical allogeneic hematopoietic stem cell transplantation; UCBT, umbilical cord blood transplantation; MSD, matched sibling donor; iTregs, induced regulatory T cells; GVHD, graft versus host disease; cGVHD, chronic graft versus host disease.

### Tregs to Prevent GVHD

A clinical trial showed that early adoptive transfer of Tregs freshly isolated from donors, followed by conventional T cells (Tcons) four days later, could prevent GVHD without increasing infection or relapse following haploidentical HSCT, which confirmed the prophylactic efficacy of Tregs against GVHD for the first time (82). Furthermore, Brunstein et al. also confirmed the safety of umbilical Tregs expanded *in vitro* using IL-2 and *α*CD3/*α*CD28 beads to prevent aGVHD after umbilical cord blood (UCB) transplantation without increasing the likelihood of infection, relapse, or early mortality (83). This study included 23 patients who received UCB-derived Tregs at a dose of 0.1–30 × 10^5^ UCB Tregs/kg on day one after transplantation, alongside a cohort receiving a second dose of 30 × 10^5^ Tregs/kg at day +15, and compared with 108 historical controls, the incidence rates of grade II–IV aGVHD were reduced by 43 and 61%, respectively (P = 0.05). A clinical trial of *in vivo* Treg expansion by injection of ultra-low dose IL-2 also reduced the incidence of GVHD (111); 16 patients were administered with ultra-low doses of IL-2 (100,000 units subcutaneously × 3 weekly for 6–12 weeks) after allo-HSCT. The percentage of Tregs increased after IL-2 therapy, with the mean of 4.8% rising to 11.1%, and no IL-2 treated patients suffered from grade II–IV aGVHD, compared with 12% (4/33) of the control group who did not receive IL-2. Besides, Pierini et al. (18) conducted a clinical trial which included 50 patients with acute myeloid leukemia (AML). An age-adapted myeloablative conditioning regimen was combined with Treg/Tcon adoptive immunotherapy, resulting in an impressive 75% moderate/severe cGVHD/relapse-free survival rate. Only two of the 50 patients relapsed, and GVL was not impaired by this therapy with Tregs, which may be related to the low levels of CXCR4 bone marrow homing receptor in Tregs.

### Tregs to Treat GVHD

Notably, no large-scale trial of Treg administration to treat GVHD, particularly aGVHD, has been conducted. The first case of cGVHD patients to receive Treg infusion reported an improved outcome, but this was not conclusively demonstrated in patients with aGVHD (15). In another clinical trial, Tregs from HLA-matched donors were administered to five patients with treatment-refractory cGVHD; two patients achieved symptomatic relief, while four had reduced immunosuppressive treatment with increased numbers of Tregs *in vivo*; however, two patients were diagnosed with skin cancer months after adoptive transfer of Tregs (112). Further studies are needed to evaluate the feasibility of this approach for treating GVHD. Regarding Treg infusion to treat patients with aGVHD, related reports are rare; however, the therapeutic efficacy of Tregs in established aGVHD has been investigated in a mouse model (113). The severity of tissue damage caused by aGVHD was alleviated and Tregs migrated to aGVHD targeted organs and lymphoid sites to exert immunosuppressive function, particularly in the gastrointestinal tract. Mice treated with Tregs showed relief of aGVHD symptoms and achieved prolonged survival (113). These results suggest that adoptive transfer of Tregs to treat aGVHD may be effective, although more studies are required.

### Tregs Combined With DLI

Previous investigations have focused on the feasibility and safety of Tregs’ transfer to control GVHD at the time of transplantation. As an effective strategy to rescue patients with relapsed hematological malignancies after allo-HSCT, donor lymphocyte infusion (DLI) can boost GVL to eliminate tumor cells, but increases the risk of severe GVHD (114). Recently, Di Ianni et al. (115) reported the case of a patient with acute promyelocytic leukemia (APL) treated with Treg-protected DLI in the early stage of recurrence after second allo-HSCT. The patient received a first infusion dose of 2.5 × 10^6^/kg Tregs, followed by infusion of 5 × 10^6^/kg Tcons one week later; a second infusion (2.5 × 10^6^/kg Tregs and 2 × 10^6^/kg Tcons) was performed two months later. The results demonstrated that complete hematological remission was achieved, with a progression-free survival of 6 months, and no apparent GVHD symptoms were observed. Whether Tregs can be administered with DLI to prevent GVHD deserves more investigation, and this is a potential new strategy to apply Tregs for GVHD control.

### Tregs and Immunosuppressive Agents

Since Tregs play a vital role in maintaining peripheral immune tolerance, pharmacological immunosuppression for GVHD prophylaxis and treatment may influence the regulation of Tregs after allo-HSCT. As hypomethylating agents, azacitidine (AzaC) and decitabine (Dec) can cause CD4^+^CD25^–^ T cells to obtain suppressive properties by inducing FOXP3 expression (116). Thus, the effect of AzaC in mitigating GVHD symptoms without abrogating GVL involves the conversion of alloreactive T cells to suppress Tregs (116). Further, nTregs are essential for AzaC protection against GVHD, as depletion of nTregs *in vivo* in mice compromised the effect of AzaC (117). The GVHD prophylactic regimen based on sirolimus without calcineurin inhibitors in patients with high-risk hematological malignancies can promote Treg proliferation and accelerate immune reconstitution *in vivo* (118). Low-dose post-transplant cyclophosphamide could attenuate and prevent GVHD by increasing Treg frequency, and the effects were enhanced by combination with anti-thymocyte globulin (119). Lee et al. (120) applied metformin and tacrolimus to treat GVHD in mice and found that combined therapy with these two drugs suppressed type 1 helper T (Th1) and Th17 cell development, while it enhanced the expression of Treg-related genes. This combination therapy alleviated GVHD severity and improved mouse survival. Furthermore, combination treatment also reduced human alloreactive T cell proliferation and production of proinflammatory cytokines by balancing the Treg/Th17 cells ratio (120). Understanding the effects of pharmaceutical preparations on Tregs could help clinicians to formulate optimal regimens to generate equilibrium between relapse and GVHD, thus achieving immune homeostasis.

## Discussion

Treg administration for GVHD has been investigated for several years. Numerous animal studies have confirmed that Tregs have important roles in restraining excessive immune responses and can prevent GVHD, without increasing the risk of relapse and infection. Tregs may lose their immunosuppressive phenotype due to unstable expression of FOXP3 under inflammatory conditions. Notably, upstream factors that regulate Tregs’ stability have not been clearly elucidated; therefore strategies to maintain FOXP3 stability warrant investigation to ensure that Tregs exert a suppressive function after adoptive transfer. The numbers of Tregs available in peripheral blood samples are far from sufficient for clinical application. In addition to exploring methods to effectively freshly isolate high purity Tregs from donors, methods for generating large numbers of functional iTregs *in vitro* deserve more attention, given the intrinsically unstable properties of Tregs cultured *ex vivo*. Notably, alloantigen-specific Tregs may possess potent inhibitory function against specific tissues, facilitating achievement of satisfactory suppressive effects using fewer cells. Furthermore, universal Tregs may broaden the utility of Treg infusion, opening a new avenue to resolution of the problem of GVHD after allo-HSCT. Many clinical trials have demonstrated that adoptive transfer of Tregs is an effective and safe way to prevent GVHD, rather than treating GVHD after it occurs. More trials are needed to verify the feasibility of cell therapy based on Treg infusion to treat GVHD, particularly aGVHD.

## Author Contributions

W-WG, X-HS and M-YW wrote the manuscript. X-MF, M-ZH and E-LJ revised the manuscript. All authors contributed to the article and approved the submitted version. W-WG and X-HS contributed equally to this manuscript.

## Funding

This work was supported by grants from National Natural Science Foundation of China (No. 81670171 and 82070192).

## Conflict of Interest

The authors declare that the research was conducted in the absence of any commercial or financial relationships that could be construed as a potential conflict of interest.
